# Supplementary material for: Content-rich biological network constructed by mining PubMed abstracts
Source: BMC Bioinformatics. 2004 Oct 8;5:147. doi: 10.1186/1471-2105-5-147 (PMC528731; doi:10.1186/1471-2105-5-147)
Supplement: Additional File 2 — The original results of the above study (non-essential files are deleted to keep the file size under the limit set by BMC bioinformatics). [file 1471-2105-5-147-S2.bz2 › chilibotAdditionalFile2/dip50/321ID10576729E2442/html/ATPH_ATPE.html]

 


 **ATPH** and **ATPE** 
  
Found 205 abstracts in PubMed, retrieved 50.  
 

 What does Google say? 
 PDF only 
| .edu only 

---

**Interactive relationship** (e.g. stimulation, inhibition, etc)

**Neutral relationship**- Adipocyte lipid binding protein  [ **ATPE** ]   [ **ATPH** ]  ALBP or aP2 is an intracellular fatty acid binding protein which is found in adipocytes and macrophages and binds a large variety of intracellular lipids with high affinity.  Ref: 12794068 J Biol Chem< MedlineTA>J Biol Chem,
- Brain lipid binding protein  [ **ATPE** ]   [ **ATPH** ]  in axon Schwann cell interactions and peripheral nerve tumorigenesis.  Ref: 12612091 Mol Cell Biol, 2003
- This has led to important insights into its function, including the interaction of HSL with other intracellular proteins, such as adipocyte lipid binding protein  [ **ATPE** ]   [ **ATPH** ] .  Ref: 12364542 J Lipid Res, 2002
- HSL has been shown to interact with adipocyte lipid binding protein  [ **ATPE** ]   [ **ATPH** ]  ALBP, a member of the family of intracellular lipid binding proteins that bind fatty acids and other hydrophobic ligands.  Ref: 11682468 J Biol Chem, 2001
- Characterization of the functional interaction of adipocyte lipid binding protein  [ **ATPE** ]   [ **ATPH** ]  with hormone sensitive lipase.  Ref: 11682468 J Biol Chem, 2001

**Non-interactive relationship** (e.g. studied together, co-existance, homology, etc.)

- Ileal ornithine decarboxylase mRNA expression was increased with PRED, but there were no steroid associated changes in the expression of the mRNA of the early response genes c myc, c jun, or c fos or of proglucagon, the liver fatty acid binding protein FABP, the ileal lipid binding protein  [ **ATPE** ]   [ **ATPH** ] , tumor necrosis factor alpha, interleukin 2 IL 2, IL 6, or IL 10.  Ref: 11911117 Lipids, 2002
- Cellular retinoic acid binding protein II CRABP II is an intracellular lipid binding protein  [ **ATPE** ]   [ **ATPH** ]  that associates with retinoic acid with a subnanomolar affinity.  Ref: 11909957 Mol Cell Biol, 2002
- Results Resection had no effect on the mRNA expression for the early response genes, for proglucagon, or for the ileal lipid binding protein  [ **ATPE** ]   [ **ATPH** ]  ILBP, but was associated with reduced jejunal ornithine decarboxylase ODC mRNA and with reduced jejunal mRNA for the liver fatty acid binding protein L FABP.  Ref: 12428071 Digestion, 2002
- heart H, brain B, myelin M, adipocyte A, epidermal E, intestinal I, liver L and ileal lipid binding protein  [ **ATPE** ]   [ **ATPH** ]  I LBP, we studied their ligand specificity, their conformational stability and their immunological crossreactivity.  Ref: 11461829 Int J Biochem Cell Biol, 2001
- The global fold of the protein is consistent with the common motif described for members of the intracellular lipid binding protein  [ **ATPE** ]   [ **ATPH** ]  family.  Ref: 11934897 J Biol Chem, 2002
- In the liver, dietary t10, c12 CLA triggered the ectopic production of peroxisome proliferator activated receptor gamma PPARgamma, adipocyte lipid binding protein  [ **ATPE** ]   [ **ATPH** ]  and fatty acid transporter mRNAs and induced expression of the sterol responsive element binding protein 1a and fatty acid synthase genes.  Ref: 12235171 J Lipid Res, 2002
- This is a 136 residue, 10 stranded, antiparallel beta barrel protein with seven beta hairpins and is a member of the intracellular lipid binding protein  [ **ATPE** ]   [ **ATPH** ]  iLBP family.  Ref: 12834350 Biochemistry, 2003
- The relative ligand binding preference of the murine ileal lipid binding protein  [ **ATPE** ]   [ **ATPH** ] .  Ref: 12651103 Protein Expr Purif, 2003
- In parallel, a rapid and dramatic decrease in the level of mRNA encoding for several adipocyte specific proteins such as adipocyte lipid binding protein  [ **ATPE** ]   [ **ATPH** ] , hormone sensitive lipase, lipoprotein lipase, and peroxisome proliferation activating receptor gamma2 was observed in isolated adipocytes.  Ref: 12734776 Horm Metab Res, 2003
- Two fatty acid binding proteins FABPs are expressed in adipose tissue, adipocyte lipid binding protein  [ **ATPE** ]   [ **ATPH** ]  ALBP and keratinocyte lipid binding protein  [ **ATPE** ]   [ **ATPH** ]  KLBP.  Ref: 12479574 Mol Cell Biochem, 2002
- Intestinal fatty acid binding protein IFABP is a member of the lipid binding protein  [ **ATPE** ]   [ **ATPH** ]  family, members of which have a clam shell type of motif formed by two five stranded beta sheets.  Ref: 11284675 Biochemistry, 2001
- Insights into the bile acid transportation system the human ileal lipid binding protein  [ **ATPE** ]   [ **ATPH** ]  cholyltaurine complex and its comparison with homologous structures.  Ref: 12486725 Proteins, 2003
- Three different classes of small lipid binding protein  [ **ATPE** ]   [ **ATPH** ]  LBP are found in helminth parasites.  Ref: 12022874 Biochemistry, 2002
- The small intestine contains three distinct proteins belonging to the intracellular lipid binding protein  [ **ATPE** ]   [ **ATPH** ]  family the liver type fatty acid binding protein L FABP, the intestinal fatty acid binding protein I FABP and the ileal lipid binding protein  [ **ATPE** ]   [ **ATPH** ]  ilbp.  Ref: 12479571 Mol Cell Biochem, 2002
- The ileal lipid binding protein  [ **ATPE** ]   [ **ATPH** ]  ILBP, a member of the intracellular lipid binding protein  [ **ATPE** ]   [ **ATPH** ]  family, is a 14 kDa protein that has bile and fatty acids as possible physiological ligands.  Ref: 12651103 Protein Expr Purif, 2003
- The present work focuses on two very different LBPs, heart type fatty acid binding protein H FABP and ileal lipid binding protein  [ **ATPE** ]   [ **ATPH** ]  ILBP.  Ref: 12237460 Protein Sci, 2002
- We have also shown that the adipocyte lipid binding protein  [ **ATPE** ]   [ **ATPH** ]  ALBP, the keratinocyte lipid binding protein  [ **ATPE** ]   [ **ATPH** ]  KLBP and the acyl CoA binding protein ACBP exhibit a prominent nuclear localization in differentiating 3T3 L1 adipocytes.  Ref: 12479581 Mol Cell Biochem, 2002
- Each protein produced a characteristic shift in peak fluorescence emission of DAUDA, and one Ce FAR 5 produced a shift greater than has been observed previously for any lipid binding protein  [ **ATPE** ]   [ **ATPH** ] .  Ref: 12502713 J Biol Chem, 2003
- These membranes are then incubated with a lipid binding protein  [ **ATPE** ]   [ **ATPH** ]  possessing an epitope tag.  Ref: 11972359 Sci STKE, 2002
- First, Notch1 activation by neuronal contact induces the glial expression of the brain lipid binding protein  [ **ATPE** ]   [ **ATPH** ]  BLBP and erbB2 genes.  Ref: 12853432 J Neurosci, 2003
- The adipocyte lipid binding protein  [ **ATPE** ]   [ **ATPH** ]  ALBP aP2 gene facilitates foam cell formation in human THP 1 macrophages.  Ref: 12417276 Atherosclerosis, 2002
- Reabsorption of bile acids occurs in the terminal ileum by a Na dependent transport system composed of several subunits of the ileal bile acid transporter IBAT and the ileal lipid binding protein  [ **ATPE** ]   [ **ATPH** ] .  Ref: 11447228 J Biol Chem, 2001
- PPARgamma protein level was decreased in a time and concentration dependent manner, and DHEA significantly reduced mRNA levels of PPARgamma, adipocyte lipid binding protein  [ **ATPE** ]   [ **ATPH** ] , and sterol regulatory element binding protein, but not CCAAT enhancer binding protein alpha.  Ref: 12488352 Endocrinology, 2003
- Among a series of adipocyte transcripts, only the adipocyte lipid binding protein  [ **ATPE** ]   [ **ATPH** ]  mRNA is also increased by DHA, although later than the PEPCK mRNA and at a much lower extent.  Ref: 11968005 J Cell Biochem, 2002
- The zebrafish CRABP exhibits highest sequence identity to the pufferfish CRABPIIa 83% and CRABPIIb 79%, and human CRABPII 74% than to any other member of the intracellular lipid binding protein  [ **ATPE** ]   [ **ATPH** ]  ILBP family.  Ref: 12853146 Gene, 2003
- Apolipoprotein E apoE is a lipid binding protein  [ **ATPE** ]   [ **ATPH** ]  that plays an important role in tissue repair following brain injury.  Ref: 12793762 J Radiat Res Tokyo , 2002
- Apolipoprotein E, the major brain lipid binding protein  [ **ATPE** ]   [ **ATPH** ] , is expressed in humans as three common isoforms E2, E3 and E4.  Ref: 11113336 Neuroscience, 2000
- Role of adipocyte lipid binding protein  [ **ATPE** ]   [ **ATPH** ]  ALBP and acyl coA binding protein ACBP in PPAR mediated transactivation.  Ref: 12479581 Mol Cell Biochem, 2002
- Members of the phylogenetically related intracellular lipid binding protein  [ **ATPE** ]   [ **ATPH** ]  iLBP are characterized by a highly conserved tertiary structure, but reveal distinct binding preferences with regard to ligand structure and conformation, when binding is assessed by the Lipidex method removal of unbound ligand by hydrophobic polymer or by isothermal titration calorimetry, a true equilibrium method.  Ref: 12479567 Mol Cell Biochem, 2002
- Taken together, these results demonstrate that Bm FAB 1 is a member of an unusual nematode specific, secreted lipid binding protein  [ **ATPE** ]   [ **ATPH** ]  family.  Ref: 12387845 Mol Biochem Parasitol< MedlineTA>Mol Biochem Parasitol, 2002
- From this analysis, we have identified adipocyte lipid binding protein  [ **ATPE** ]   [ **ATPH** ]  ALBP aP2 as a gene that is highly upregulated in foam cells in response to oxLDL.  Ref: 12417276 Atherosclerosis, 2002
- Treating differentiating 3T3 L1 preadipocytes with t10c12 CLA and conjugated nonadecadienoic acid CNA, a 19 carbon CLA cognate resulted in decreased intracellular triglyceride accumulation and mRNA levels of the adipogenic gene fatty acid synthase and adipocyte lipid binding protein  [ **ATPE** ]   [ **ATPH** ] .  Ref: 12670481 Biochem Biophys Res Commun, 2003
- The binding specificity of the protein is not known based on the high sequence and structural similarity with an orthologous protein, ileal lipid binding protein  [ **ATPE** ]   [ **ATPH** ] , it is suggested that bile acids may be the putative ligands.  Ref: 12652125 J Biomol NMR, 2003
- No genes for photosynthetic functions have been found, except for sequences encoding six subunits of the ATP synthase atpA, atpB,  **atpE** , atpF,  **atpH** , and atpI.  Ref: 12111556 Mol Genet Genomics, 2002
- Using cDNA microarrays, we found that brain lipid binding protein  [ **ATPE** ]   [ **ATPH** ]  BLBP is elevated in an EGFR positive subpopulation of Nf1 mutant mouse Schwann cells Nf1 TXF that grows away from axons.  Ref: 12612091 Mol Cell Biol, 2003
- The adipocyte and heart fatty acid binding proteins A and HFABP are members of a lipid binding protein  [ **ATPE** ]   [ **ATPH** ]  family with a beta barrel body capped by a small helix turn helix motif.  Ref: 11675390 J Biol Chem, 2002
- To determine whether NS5A interacts with any cellular lipid binding protein  [ **ATPE** ]   [ **ATPH** ] , we performed yeast two hybrid screening in both HepG2 and human liver cDNA libraries.  Ref: 11878923 Virology, 2002
- Salt modulates the stability and lipid binding affinity of the adipocyte lipid binding protein  [ **ATPE** ]   [ **ATPH** ] .  Ref: 12794068 J Biol Chem< MedlineTA>J Biol Chem, 2002
- Among fatty acids, mILBP preferred species that had longer chain length and increased saturation, similar to other members of the intracellular lipid binding protein  [ **ATPE** ]   [ **ATPH** ]  family.  Ref: 12651103 Protein Expr Purif, 2003
- We found that the C2 domain a calcium sensing, lipid binding protein  [ **ATPE** ]   [ **ATPH** ]  module that is an essential regulator of protein kinase C and numerous other proteins targeted proteins to the plasma membrane transiently if calcium was released from internal stores, and persistently in response to entry of extracellular calcium across the plasma membrane.  Ref: 11884760 Science, 2002
- Most of these cells express vimentin and brain lipid binding protein  [ **ATPE** ]   [ **ATPH** ] .  Ref: 12088755 Neuroscience, 2002
- Ontogenetic development and spatial distribution of the ileal apical sodium dependent bile acid transporter and the ileal lipid binding protein  [ **ATPE** ]   [ **ATPH** ]  in apoE knockout and C57BL 6 mice.  Ref: 12374235 Scand J Gastroenterol, 2002
- Adipose specific fatty acid binding protein aP2 and keratinocyte lipid binding protein  [ **ATPE** ]   [ **ATPH** ]  abundance was higher in differentiated rat perirenal than in epididymal preadipocytes.  Ref: 11158926 Am J Physiol Endocrinol Metab, 2001
- In fully differentiated 3T3 L1 adipocytes, TZDs markedly decreased PPARgamma mRNA levels without reducing the expression of genes that are positively regulated by PPARgamma, such as adipocyte lipid binding protein  [ **ATPE** ]   [ **ATPH** ]  2 aP2 or lipoprotein lipase LPL.  Ref: 11172472 Metabolism, 2001
- METHODS Adipocyte lipid binding protein  [ **ATPE** ]   [ **ATPH** ]  ALBP and keratinocyte lipid binding protein  [ **ATPE** ]   [ **ATPH** ]  KLBP expression was quantified by western blot in subcutaneous and omental adipose tissue from 20 obese and 9 lean individuals.  Ref: 11692175 Diabetologia, 2001
- For example, it has been reported that some beta proteins in the intracellular lipid binding protein  [ **ATPE** ]   [ **ATPH** ]  family fold through quite different pathways Burns et al.  Ref: 12784211 Proteins, 2003
- To investigate the role of ERK activation, co treatment of RA with PD98059, a specific inhibitor of the ERK signalling pathway, prevented both adipocyte formation and expression of the adipogenic markers, adipocyte lipid binding protein  [ **ATPE** ]   [ **ATPH** ]  and peroxisome proliferator activated receptor gamma.  Ref: 11802792 Biochem J, 2002
- Moreover, phytanic acid activates brown adipocyte differentiation long term exposure of brown preadipocytes to phytanic acid promoted the acquisition of the brown adipocyte morphology and caused a co ordinate induction of the mRNAs for gene markers of brown adipocyte differentiation, such as UCP1, adipocyte lipid binding protein  [ **ATPE** ]   [ **ATPH** ]  aP2, lipoprotein lipase, the glucose transporter GLUT4 or subunit II of cytochrome c oxidase.  Ref: 11829740 Biochem J, 2002
- The procedures developed will be useful for sequencing other oligosaccharides including those from other members of the lipid binding protein  [ **ATPE** ]   [ **ATPH** ]  class to which cerebroside sulfate activator belongs.  Ref: 11180632 J Mass Spectrom, 2000
- These genes were peripheral myelin protein 22, decorin, transcription factor AP 1, dystroglycan 1, myelin protein zero, mitogen activated protein kinase 3, prothymosin beta 4, and brain lipid binding protein  [ **ATPE** ]   [ **ATPH** ] .  Ref: 12036991 Invest Ophthalmol Vis Sci, 2002
- Here we report the solution structure of the human ileal lipid binding protein  [ **ATPE** ]   [ **ATPH** ]  ILBP in free form and in complex with cholyltaurine.  Ref: 12486725 Proteins, 2003
- The features of E FABP backbone dynamics elaborated in the present study differ markedly from those of the phylogenetically closely related heart type FABP and the more distantly related ileal lipid binding protein  [ **ATPE** ]   [ **ATPH** ] , implying a strong interdependence with the overall protein stability and possibly also with the ligand binding affinity for members of the lipid binding protein  [ **ATPE** ]   [ **ATPH** ]  family.  Ref: 12049637 Biochem J, 2002
- These include diminished expression of the brain lipid binding protein  [ **ATPE** ]   [ **ATPH** ] , which is required for glial morphological differentiation, and the basic helix loop helix NeuroD Beta2 and homeodomain Engrailed 2 transcription factors, which are required for normal cerebellar morphogenesis and foliation.  Ref: 11444531 Gene Expr, 2001
